# Deep Brain Stimulation Improves the Symptoms and Sensory Signs of Persistent Central Neuropathic Pain from Spinal Cord Injury: A Case Report

**DOI:** 10.3389/fnhum.2017.00177

**Published:** 2017-04-06

**Authors:** Walter J. Jermakowicz, Ian D. Hentall, Jonathan R. Jagid, Corneliu C. Luca, James Adcock, Alberto Martinez-Arizala, Eva Widerström-Noga

**Affiliations:** ^1^The Miami Project to Cure Paralysis, Miller School of Medicine, University of MiamiMiami, FL, USA; ^2^Department of Neurological Surgery, Miller School of Medicine, University of MiamiMiami, FL, USA; ^3^Research Service, Bruce W. Carter Department of Veterans Affairs Medical CenterMiami, FL, USA; ^4^Department of Neurology, Miller School of Medicine, University of MiamiMiami, FL, USA

**Keywords:** neuromodulation, low-frequency stimulation, periaqueductal gray, pain severity, evoked pain, chronic pain

## Abstract

Central neuropathic pain (CNP) is a significant problem after spinal cord injury (SCI). Pharmacological and non-pharmacological approaches may reduce the severity, but relief is rarely substantial. While deep brain stimulation (DBS) has been used to treat various chronic pain types, the technique has rarely been used to attenuate CNP after SCI. Here we present the case of a 54-year-old female with incomplete paraplegia who had severe CNP in the lower limbs and buttock areas since her injury 30 years prior. She was treated with bilateral DBS of the midbrain periaqueductal gray (PAG). The effects of this stimulation on CNP characteristics, severity and pain-related sensory function were evaluated using the International SCI Pain Basic Data Set (ISCIPBDS), Neuropathic Pain Symptom Inventory (NPSI), Multidimensional Pain Inventory and Quantitative Sensory Testing before and periodically after initiation of DBS. After starting DBS treatment, weekly CNP severity ratings rapidly decreased from severe to minimal, paralleled by a substantial reduction in size of the painful area, reduced pain impact and reversal of pain-related neurological abnormalities, i.e., dynamic-mechanical and cold allodynia. She discontinued pain medication on study week 24. The improvement has been consistent. The present study expands on previous findings by providing in-depth assessments of symptoms and signs associated with CNP. The results of this study suggest that activation of endogenous pain inhibitory systems linked to the PAG can eliminate CNP in some people with SCI. More research is needed to better-select appropriate candidates for this type of therapy. We discuss the implications of these findings for understanding the brainstem’s control of chronic pain and for future progress in using analgesic DBS in the central gray.

## Introduction

Persistent neuropathic pain is a common and serious consequence of spinal cord injury (SCI) that is especially refractory to both pharmacological and non-pharmacological treatments (Siddall et al., 2003; Vranken, 2009; Finnerup et al., 2014). Electrical stimulation of specific brain structures with the purpose of relieving chronic pain has been used for a long time but the reported efficacy for central neuropathic pain (CNP) is relatively low and difficult to predict. For certain patients, effects of stimulation on CNP may be tremendous (Boccard et al., 2013; Pereira and Aziz, 2014). Nevertheless, because deep brain stimulation (DBS) is an invasive procedure, its use for SCI pain is rare and currently restricted to severe cases refractory to non-invasive therapies. In order to be accepted as a therapy for chronic pain, success rates need to improve. This will require, *inter alia*, a better understanding of CNP mechanisms along with improved patient selection.

The present article describes an almost complete reversal of CNP (which had been present since injury 30 years ago) and associated sensory dysfunction paralleled by a reduction in psychosocial impact in a 54-year-old female with SCI-related CNP who underwent DBS of the periaqueductal gray (PAG). The optimization of stimulation parameters and the time-course of daily changes in general pain intensity over a 42-week period were previously reported for this subject and one other with SCI-related chronic pain (Hentall et al., 2016). Here we provide a more detailed analysis specifically focusing on the CNP below the level of injury evaluating a broader set of pain characteristics, somatosensory function and psychosocial impact conducted at intervals over a 52-week period. We also conducted an exit interview to obtain her personal perspectives on her pain and treatments. The “Discussion” Section contains a brief review of possible mechanisms underlying successful amelioration of CNP by DBS and the prospects for improving treatment efficacy.

## Background

### Clinical History

The subject was a 54-year-old female veteran with an incomplete SCI (T11 level) due to electrocution 30 years ago. Examination according to the International Standards for Neurological Classification of SCI classified her injury as AIS-B (sensory but no motor function preserved).

The patient’s worst pain problem was chronic below-level CNP in both lower extremities, with onset shortly after her initial injury. The average weekly pain level for this specific pain preceding the baseline measurement was 8 of 10 on a numerical rating scale (NRS). CNP was daily and constant, and most intense during the night and therefore significantly interfering with sleep. She reported severe “electric” and moderate “stabbing” pain, moderate to severe evoked pain in response to brushing, severe evoked pain to cold stimuli and moderate tingling and pins and needles in the painful area. She was taking a daily dose of 75 mg pregabalin for the pain but had previously tried a wide range of pharmacological options (including opiates and higher doses of pregabalin) and she said: “Medication? I don’t like medication. I don’t like the way it makes me feel. I don’t like the side effects. It … They all leave me feeling stupid and foggy and disoriented and … So, that’s why I wouldn’t take them until I absolutely just couldn’t …”

### Pain and Sensory Assessments

**Figure 1:**
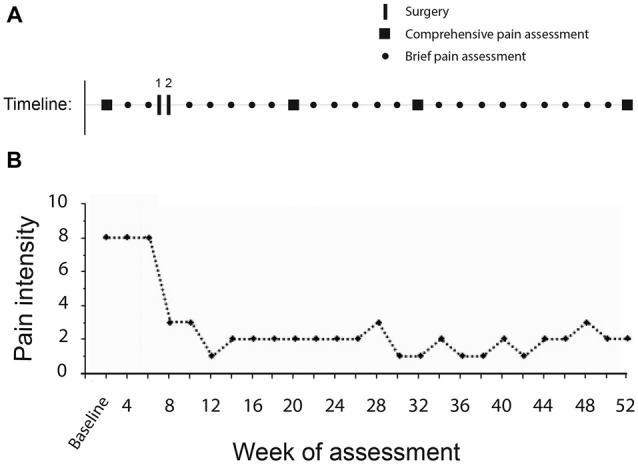
**(A)** Timeline of study. The subject underwent a comprehensive pain assessment (entire battery of pain tests) on week 2, prior to the two surgical procedures performed on weeks 6 and 7. This was followed by biweekly brief pain assessments (average pain intensity ratings past 7 days by patient in clinic) and periodic repeat comprehensive assessments postoperatively (weeks 20, 32 and 52). **(B)** Average pain intensity ratings past 7 days through duration of the study. Immediately following activation of the device the subject noticed a profound reduction of her central neuropathic pain (CNP).

Pain was evaluated with respect to location, classification, intensity, temporal pattern and pain interference using the International SCI Pain Basic Dataset (ISCIPBDS; Widerström-Noga et al., 2014). Neuropathic pain symptom severity was evaluated with the Neuropathic Pain Symptom Inventory (NPSI; Bouhassira et al., 2004), and psychosocial impact with the Multidimensional Pain Inventory-SCI version (Widerström-Noga et al., 2006). Sensory function was assessed below the level of injury in the neuropathic pain area with the TSA-II Neurosensory Analyzer (Medoc Ltd., Ramat Yishai, Israel), either via a thermode or a vibratory pin applied to the painful area below the level of injury. Thresholds for cool detection, warm detection, cold pain, hot pain and vibration detection were determined using the method of limits (reaction-time inclusive). We also evaluated thermal allodynia using thermorollers (Somedic, Sweden), and mechanical allodynia using a soft brush and Semmes-Weinstein monofilaments (10 g, #5.07). Further details regarding all QST procedures can be found in a previous publication (Widerström-Noga et al., 2016). Baseline pain testing was performed twice in the subject (1 and 5 weeks before the first surgery) and baseline results were averaged. For a study timeline see Figure 1.

### DBS Surgery

The work was performed under an Investigative Device Exemption of the U.S. Food and Drug Administration (IDE G120202), and Clinical Trials.gov (NCT02006433). All study procedures were approved by the University of Miami Institutional Review Board (IRB) with written consent from the subject in accordance with the Declaration of Helsinki.

Two surgeries were performed 1 week apart: (1) bilateral implantation of electrode leads (Medtronic3387S-40) in the anterolateral PAG with the subject awake; and (2) connection of both leads to extension cables under general anesthesia and tunneling to a generator (Activa PC Neurostimulator 37601, Medtronic). Stimulation was briefly tested during the first surgery, eliciting an emotional response in the subject due to near-complete relief of her long-standing symptoms. For more details see Hentall et al. (2016).

## Results

The subject reported a rapid and profound improvement in her CNP, from severe to minimal intensity, when the DBS device was activated on the day after the second surgery. Her DBS stimulation settings were adjusted in periodic office visits (monthly, later every 2 months). In addition, the stimulator was programmed with several blinded choices of parameters (frequency or voltage) for selection at home (Hentall et al., 2016). Among salient findings reported previously, she preferred a very low mean pulse rate (0.67 Hz) and the pain level took several days to shift to a new steady state when the blinded stimulation setting was changed. The blinded choice of settings (ineffective 0.1 V vs. effective 4.5 V) also allowed exclusion of potentially powerful placebo effects.

**Figure 2:**
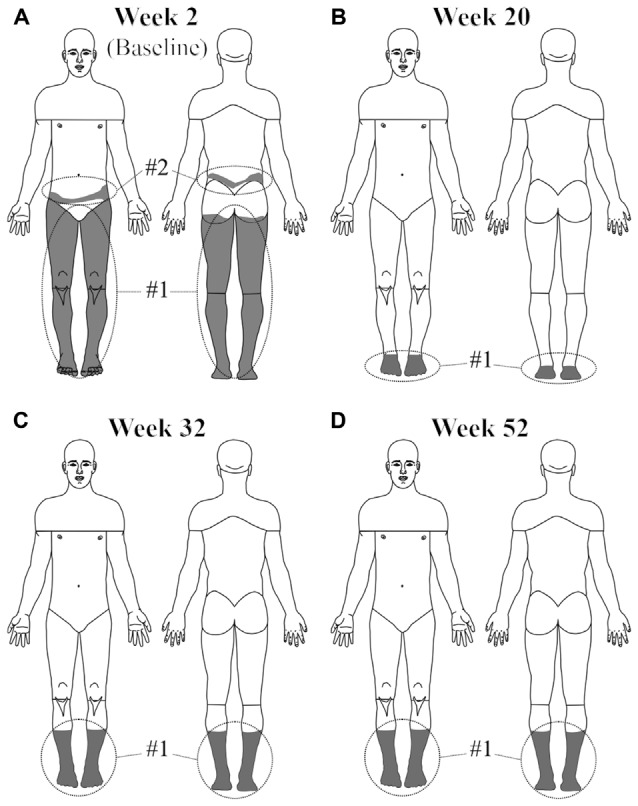
**The area of neuropathic pain was greatly reduced in the subject with activation of the device.** Whereas before surgery **(A)** her pain was located at the anterior and posterior aspects of both legs and the superior buttocks, by the 20th week after surgery **(B)** pain was only perceived below the ankles. By the 32nd postoperative week **(C)** the pain extended back up to her mid-calf region of both legs and remained unchanged the remainder of the study **(D)**.

Figure 1B shows a time-line of the subject’s average weekly CNP intensity following her DBS procedure. Whereas she typically reported a pain intensity of 7–8 at baseline, following the procedure her median reported CNP intensity was 2 and remained so the length of the study. The subject also noted a significant decrease in the size of the painful area (Figure 2). Pain medication was discontinued by the 17th postoperative week.

**Figure 3:**
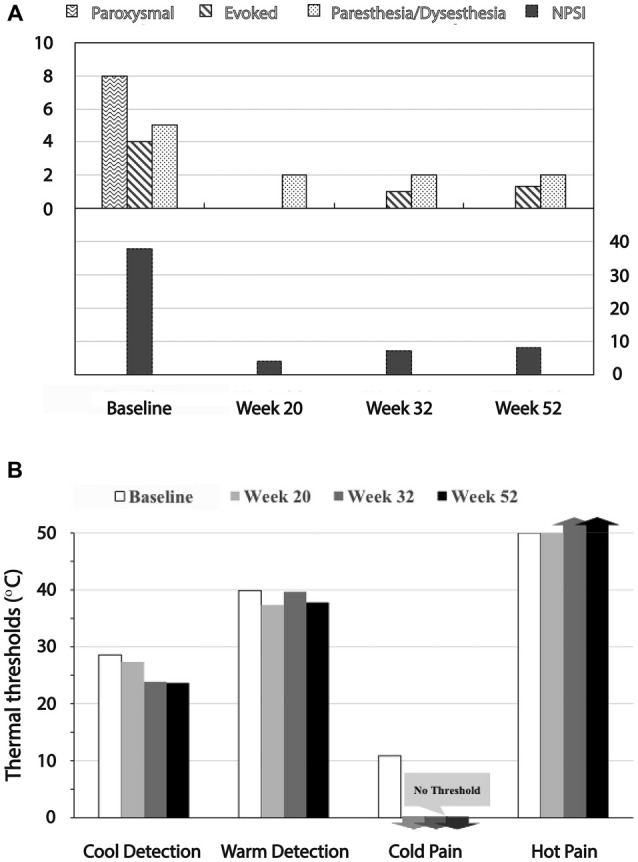
**Reduction of Neuropathic Pain Symptom Inventory (NPSI) and QST test scores with device activation. (A)** The top graph shows paroxysmal, evoked and paresthesia/dysesthesia scores, all of which were reduced to minimal levels with stimulation. The bottom graph shows the reduction in the NPSI sum score. **(B)** Effects on QST measures. Whereas the subject had significant allodynia before surgery in response to cold and warm, these stimuli within the default minimum/maximum temperatures (0°C and 50°C, respectively) did not evoke pain sensations in follow-up.

The rapid reduction in overall neuropathic pain symptom severity after initiation of DBS was maintained over the course of the study (Figure 3A). Evoked pain was reduced to a minimal level. Paresthesia/dysesthesia (pins and needles and tingling) in the painful area also decreased to a significantly lower level.

Before surgery, the subject exhibited both dynamic mechanical allodynia evoked by very weak vibratory stimuli and static mechanical allodynia in the painful area in response to punctuate stimuli applied with the von Frey filament (100 g). Cold allodynia was evoked within a normally non-noxious range, between 22.9°C and 25.2°C. After onset of DBS treatment, however, there were no signs of allodynia in the painful area, consistent with the reduction in pain symptoms. Vibratory stimuli at the threshold level for perception and the punctuate stimulus applied with the large von Frey filament (300 g; 6.65) did not evoke pain after DBS was initiated. Cool and warm detection thresholds were in the normal range (Figure 3B). Although she initially perceived pain at the maximal temperature (50°C), both cold and hot stimuli within the default minimum/maximum temperatures of 0°C and 50°C did not evoke a pain sensation at follow-up.

Though formal neurocognitive evaluation was not performed, the Beck depression inventory (BDI), MPI-SCI subscales, perceived life control and affective distress did not indicate any negative effects of the DBS (Supplementary Figure S1). The BDI scores are not shown because the subject never scored above “1” (including the baseline assessments). Indeed, placement of the PAG DBS device had a tremendous positive impact on the quality of life of the subject. During a qualitative interview after the surgery she stated “It’s a whole new world. I’m learning my body all over again. It’s life-changing. It still brings tears to my eyes”. The DBS device has remained activated with consistent efficacy reported by the patient since implantation over 2 years ago.

## Discussion

Electrical stimulation of the PAG significantly reduced the intensity and distribution of CNP-related symptoms and associated abnormal sensory signs (i.e., mechanical and thermal allodynia) in our subject and, consequently, had a profound effect on her quality of life. The consistent beneficial effects on multiple aspects of CNP over the entire study period and the blinded choice of settings controlling for potential placebo effects (Hentall et al., 2016) supports the validity of this case report. Despite 30 years of limited pain relief with various pharmacological treatments she had near-complete relief of her CNP with activation of the DBS device. Such profound and long-lasting improvements in people with CNP pain are rarely reported with conservative measures or other surgical options (Vranken, 2009; Cardenas et al., 2013; Gao et al., 2016).

### DBS for Neuropathic Pain

DBS has been used for the treatment of various types of pain since the 1970s, following advances in implantable stimulation devices and the influence of Melzack and Wall’s (1965) gate theory. Most evidence available supports the PAG as a useful stimulation target (Pereira and Aziz, 2014). In addition to ascending somatosensory projections, the PAG receives significant inputs from the prefrontal cortex (PFC) and amygdala and then projects to nucleus raphe magnus (NRM) and locus coeruleus (LC) in the midbrain and dorsal horn neurons in the spinal cord (Van Bockstaele et al., 1991; Li et al., 2016). Recent case series on DBS for chronic neuropathic pain, which have utilized superior targeting methods compared to earlier studies and multi-day trials with an externalized electrode prior to final implantation, suggest clinically-relevant improvements of pain symptoms in 67%–83% of patients with heterogeneous types of pain (Boccard et al., 2013). Gray et al. (2014) showed that beyond improving pain symptoms, DBS may also improve mood, anxiety and quality of life. However, very few studies have examined the pain reducing ability of DBS in people with CNP. The acceptance of this therapy in the U.S. has been hindered by the variability of outcomes. Even in cases where patients respond favorably to a several day trial with an externalized stimulator prior to permanent implantation, roughly a quarter of those patients do not experience benefit 1 year after surgery (Boccard et al., 2013).

The recent renewal of interest in DBS for chronic pain has arisen from various factors: (1) improved safety of DBS in general; (2) success with other indications for DBS; (3) advances in imaging and targeting methods; and (4) renewed recognition by the public and health administrators of the detrimental effects of prolonged opioid medication (Schofferman et al., 2014). For patients with CNP of whose etiological or pathological classification is known to make it particularly refractory to typical treatment interventions, particularly if they have failed to respond to conservative measures over many years, DBS may offer benefits. The challenge is to determine which pain phenotypes and patients are most likely to respond. This is an especially hard problem in populations with CNP stemming from CNS injuries, in which multiple pain mechanisms are likely to operate simultaneously (Carlton et al., 2009; Hari et al., 2009; Zeilig et al., 2012; Finnerup et al., 2014).

### Possible Mechanisms of DBS for CNP

Spontaneous and evoked excitability within the somatosensory system is normally well-controlled by ascending and descending pain pathways (Basbaum and Fields, 1984; Vranken, 2009). CNP after spinal injury may arise as a consequence of the amplification of signals in residual sensory neurons by spinal and supraspinal processes, such as astrocytic and microglial activation, ultimately influencing various neurotransmitter systems, local connectivity and cytokines (Scholz and Woolf, 2007; Zeilig et al., 2012; Finnerup et al., 2014; Robinson et al., 2016).

The raphe nuclei and their associated modulatory neurotransmitter, serotonin, have long been implicated as important factors influencing pain. However, although selective serotonin reuptake inhibitors are among the most prescribed medications for CNP, little efficacy has been shown in clinical pain trials (Vranken, 2009; Urtikova et al., 2012). While initial behavioral experiments suggested an anti-nociceptive role for serotonin, the link between the neurotransmitter and pain is now viewed as more complex and serotonin may have both pro- and anti-nociceptive properties, depending on the location and mechanism of release (central from raphe vs. peripheral from mast cells) and the serotonergic receptors involved (Urtikova et al., 2012; Bobinski et al., 2015).

The NRM is the largest CNS source of spinal cord serotonin and the PAG is its major input (Van Bockstaele et al., 1991). Serotonergic neurons in the raphe are responsive to inflammatory molecules and markers of CNS injury (Vanegas and Schaible, 2004; Bobinski et al., 2015). It was shown that stimulation of the NRM acutely increases levels of spinal cord PKA, cAMP and CREB, signaling molecules with important roles in development, repair and long-term potentiation. Interestingly, these beneficial effects were elicited at quite low stimulation frequencies (4–8 Hz), not the high frequencies (typically 50–150 Hz) used for nearly all other indications of DBS (Hentall et al., 2006; Carballosa-Gonzalez et al., 2014). This is relevant because while high-frequency DBS is thought to functionally inhibit its target, low-frequency stimulation is considered stimulatory and, in the present case, should lead to activation of PAG and NRM and the release of modulatory neurotransmitters in the spinal cord (Hentall and Gonzalez, 2012).

Norepinephrine (NE) has also been implicated in the modulation of pain (Hickey et al., 2014; Li et al., 2016). NE reuptake inhibitors or combinations of NE and 5-HT reuptake inhibitors are common therapies for CNP and are recommended first-line treatments for patients with SCI (Guy et al., 2016). Also, the α2 receptor agonist clonidine is used to treat the withdrawal effects of opioids (Siddall et al., 2000). Recent optogenetic studies have shown that focal activation of the LC, whose major inputs are from the PFC, amygdala, PAG and dorsal noradrenergic bundle, may be either pro- or anti-nociceptive, suggestive of different neuron subgroups within the LC (Hickey et al., 2014). Consistent with this, recently Li et al. (2016), used a canine adenoviral vector expressing channelrhodopsin2 to target ponto-spinal NE neurons, and found a subset of ventral LC neurons with spinal projections that is likely involved in the regulation of nociception. Interestingly, this subgroup of LC neurons had lower average firing rate than other noradrenergic neurons of the LC.

**Figure 4:**
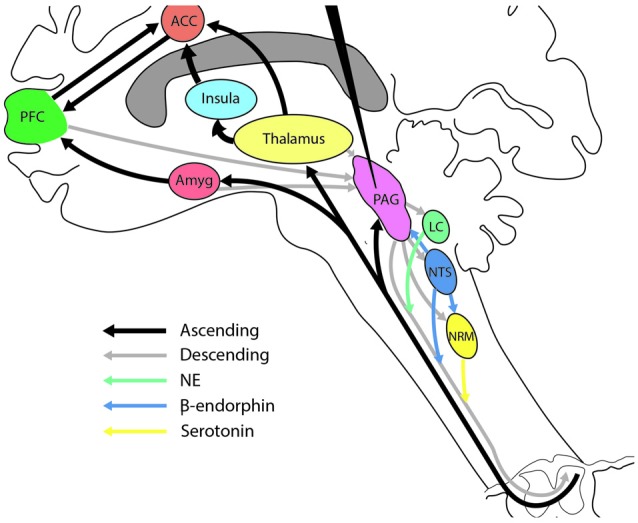
**Illustration showing the central role of the periaqueductal gray (PAG) in the modulation of various descending pain systems.** Various neurotransmitter systems have been implicated in the treatment of central pain. The PAG has strong ascending and descending connections with each of these systems and is thus in a strong position to modulate CNP. Conventions for the arrows are shown at the bottom left of the figure. ACC, anterior cingulate cortex; Amyg, amygdala; PFC, prefrontal cortex; LC, locus coeruleus; NRM, nucleus raphe magnus; NTS, nucleus tractus solitarius.

DBS of the PAG could also relieve pain via neurons that secrete β-endorphin (Siddall et al., 2000; Schofferman et al., 2014). β-endorphin is derived from the prohormone pro-opiomelanocortin (POMC). Cerritelli et al. (2016) recently identified a subset of 100–200 POMC neurons in the nucleus tractus solitarius (NTS) that, when optogenetically stimulated, led to significant analgesic effects, that were blocked by administration of naloxone, an opioid receptor antagonist. Thus several highly interconnected pathways may be involved in modulating ascending pain information and are accessible by DBS in the PAG. For a schematic overview see Figure 4.

### Prospects for Improved Rates of Success

DBS of brainstem targets can be considered for ameliorating severe CNP that is refractory to pharmacological treatment. The best evidence for any pharmacological treatment of CNP after SCI is for the anticonvulsant pregabalin (Guy et al., 2016). However, the treatment responses to pregabalin for CNP are not consistent. Indeed, a large recent randomized trial showed the numbers needed to treat for >30% pain reduction was about 7 (Cardenas et al., 2013). Long-term opioid therapy has already proven to be a poor long-term option (Schofferman et al., 2014). When available, non-invasive brain stimulation methods, such as repetitive transcranial magnetic stimulation, are preferrable over DBS. However, their efficacy is yet to be proven in heterogeneous pain conditions and they are currently not recommended treatment approaches for patients with CNP related to SCI (Gao et al., 2016; Guy et al., 2016).

If DBS is to become an accepted therapy for CNP, its efficacy and consistency need to be improved. Anecdotally patients may experience outstanding results, but many are left to a greater or lesser extent with the original debilitating pain (Coffey, 2001; Boccard et al., 2013; Pereira and Aziz, 2014). It is thus important to identify predictors of long-term efficacy and understand mechanisms of tolerance. Comprehensive evaluation of neuropathic pain symptoms including severity, location and temporal pattern, and quantitative pain assessments, such as used in this study, may help in this task, along with novel brain imaging techniques. Thus, better defined pain phenotypes based on comprehensive pain assessment protocols may lead to more reliable selection of patients amenable to this therapy. Better knowledge of responsive pain phenotypes in this notoriously difficult patient population is also important for comparing treatments, especially because novel therapies continue to emerge, such as minimally invasive ablative techniques (Tiwari et al., 2014).

## Author Contributions

WJJ performed the majority of work on preparing the manuscript, figures and integrating other authors’ comments. JA conducted pain assessments and helped prepare the pain data and figures. IDH prepared the application for the clinical trial and helped prepare the manuscript. JRJ, AM-A and CCL performed the surgical and neurological procedures and revised the final versions of the manuscript. EW-N helped prepare the manuscript and oversee and summarize all pain assessments.

## Funding

Funding for this project was provided by the U.S. Department of Defense—Spinal Cord Injury Program (W81XWH-12-1-0559).

## Conflict of Interest Statement

The authors declare that the research was conducted in the absence of any commercial or financial relationships that could be construed as a potential conflict of interest.

## Supplementary Material

FIGURE S1 **Assessment of psychosocial impact and subjective distress experienced by the patient before and after surgery.** Comparison of MPI—spinal cord injury (SCI) subscales, Life Control and Affective Distress, suggest the patient did not develop new cognitive or affective impairments following the surgery. Each assessment was performed by the same assessor. More details about the MPI-SCI may be found at Widerström-Noga et al. (2006).
