# Supplementary material for: Deep Brain Stimulation Improves the Symptoms and Sensory Signs of Persistent Central Neuropathic Pain from Spinal Cord Injury: A Case Report
Source: Front Hum Neurosci. 2017 Apr 6;11:177. doi: 10.3389/fnhum.2017.00177 (PMC5382156; doi:10.3389/fnhum.2017.00177)
Supplement: FIGURE S1 — Assessment of psychosocial impact and subjective distress experienced by the patient before and after surgery. Comparison of MPI—spinal cord injury (SCI) subscales, Life Control and Affective Distress, suggest the patient did not develop new cognitive or affective impairments following the surgery. Each assessment was performed by the same assessor. More details about the MPI-SCI may be found at Widerström-Noga et al. (2006). [file Table_1.docx]

|  | Life Control | Affective Distress |
| --- | --- | --- |
| Baseline | 1.88 | 2.00 |
| 20 weeks | 0.33 | 0.33 |
| 32 weeks | 0.00 | 1.33 |
| 52 weeks | 0.00 | 1.67 |

**Supplemental Figure 1**
